# A context-calibrated framework based on uncrewed aerial vehicle imagery for power-line detectability and mitigation evaluation: a model system from Black-necked Crane wintering grounds on the Tibetan Plateau

**DOI:** 10.7717/peerj.21732

**Published:** 2026-09-15

**Authors:** Xintong Li, Yumin Guo

**Affiliations:** College of Ecology and Nature Conservation/College of National Park, Beijing Forestry University, Beijing, China

**Keywords:** Black-necked Crane, Power transmission lines, Warning devices, Visual Complexity Index (VCI), Collision Risk Index (CRI), Collision risk assessment

## Abstract

Overhead power lines are a major source of avian mortality, yet quantitative and context-calibrated assessments of wire detectability and marker performance under variable field conditions remain scarce. Using 560 uncrewed aerial vehicle (UAV) images from three datasets collected in Black-necked Crane (*Grus nigricollis*) wintering grounds on the Tibetan Plateau, we quantified wire detectability with a Visibility Index (VI; target–background contrast and edge strength) and characterized background heterogeneity with a Visual Complexity Index (VCI; entropy, edge density, and colour variance at global and local scales). We integrate these metrics into a Collision Risk Index (CRI) as a relative, image-based proxy for detectability to support span prioritization and context-matched evaluation of mitigation devices. Within the warning-device datasets, which included sunny and cloudy conditions, sleeves provided more robust detectability gains, particularly at longer distances, whereas balls yielded smaller and less consistent improvements. Detectability varies strongly with weather, distance, and relative observation height, highlighting the need to compare mitigation options under matched contexts. We frame this high-altitude wintering landscape as a model system for collision-risk mitigation and provide practical guidance for adapting and recalibrating the workflow to other species, habitats, and linear infrastructures.

## Introduction

In recent years, the global energy sector has undergone rapid transformation, accompanied by the expansion of electricity generation and transmission infrastructure into remote, high-altitude regions, migratory corridors, and wetland areas (International Energy Agency, 2023). Such expansion has changed how energy is distributed and has had profound ecological impacts, particularly in sensitive ecological zones (Gasparatos et al., 2017). Overhead power lines have become a primary threat to bird species in regions where transmission corridors overlap with bird habitats, such as migratory paths and wintering grounds, leading to increased collision risks. This overlap threatens the survival of endangered species (Bernardino et al., 2018; Lehman, Kennedy & Savidge, 2007). From a regional sustainability perspective, this scenario exemplifies the conflict between infrastructure development and wildlife conservation in high-altitude ecosystems, underscoring the need to balance energy expansion with biodiversity protection. Assessing and mitigating potential risks to wildlife, while maintaining efficient energy transmission, has become a critical challenge in global conservation efforts (McDonald et al., 2009).

Migratory birds often concentrate in great numbers at wintering grounds (Raine et al., 2025), which increases local collision risk (Newton, 2023). For example, swans and cranes are more likely to collide with power lines in winter as they frequently traverse electrical grids (Bernardino et al., 2018). Typical winter weather conditions, such as snow, fog, and low light, impair birds’ ability to recognize power lines. This significantly increases the probability of collisions (Bevanger, 1994; Bevanger, 1998; Prinsen et al., 2011; Martin, 2011). Therefore, a multi-factor risk assessment combining visibility, habitat capacity, and bird behavior is necessary during the wintering period. This will help formulate targeted conservation strategies (NatureScot, 2025).

Birds rely heavily on vision to avoid obstacles, and visibility determines whether they can avoid man-made structures in time (Martin & Shaw, 2010; Martin, 2011). Visibility is influenced by various factors, including weather, lighting, background complexity, as well as the height, color, and arrangement of structures (Avian Power Line Interaction Committee, 2012; Renewables Grid Initiative, 2024; Marques et al., 2014; Hein, Arnett & Erickson, 2013). For large migratory birds such as the Black-necked Crane (*Grus nigricollis*), low visibility of power lines ahead can easily lead to collisions (Wang et al., 2021). Additionally, flight height alters birds’ viewing angles, presenting different visual challenges between high-altitude and low-altitude flights (Jenkins, Smallie & Diamond, 2010; Martin, 2011; Martin, 2012). Therefore, improving the visibility of structures is an effective means of reducing risk (Gauld et al., 2022; Martin, 2022). However, visual detectability should not be interpreted as a simple human-visible contrast problem. Avian perception differs from human vision in spectral sensitivity, visual-field configuration, attention, and flight behaviour, and collision risk may occur even when obstacles appear conspicuous to human observers (Martin & Shaw, 2010; Martin, 2011; Martin, 2012). Therefore, in this study, “visibility” is formally defined as the relative optical detectability of transmission lines in standard uncrewed aerial vehicle (UAV) RGB images under matched acquisition conditions. It is quantified using target–background contrast, edge strength, visible line length, and background complexity, and should not be interpreted as a direct reconstruction of Black-necked Crane spectral perception or behavioural detection thresholds.

In order to reduce bird collisions with power lines, numerous strategies have been implemented worldwide (BirdLife International, 2022; Ferrer & Janss, 1999), and the installation of line-marking devices has become a widely used mitigation option. Warning balls, spiral warning sleeves, hanging ribbons, and flashing devices can increase visual salience under some conditions (Martin & Shaw, 2010; Morkill & Anderson, 1991). Previous mitigation studies have provided important evidence on the efficacy of specific marker types, including bird-flight diverters and ultraviolet illumination, but their primary focus has often been on overall mortality reduction or device performance rather than on explicitly quantifying detectability across matched combinations of weather, viewing distance, flight or observation height, and viewing angle (Ferrer et al., 2020; Baasch et al., 2022). This leaves a practical gap: managers still lack a standardized image-based workflow for comparing mitigation options under the same environmental and geometric contexts. In this framework, the broad visibility drivers described above were translated into measurable UAV-image variables. Weather and lighting were treated as acquisition contexts, whereas observation distance, viewing angle, and flight/observation height represented viewing geometry. The optical conspicuity of the target wire was quantified using VI through target–background contrast and edge strength, while surrounding background heterogeneity was quantified using the Visual Complexity Index (VCI) through entropy, edge density, and colour variance. Thus, the Visibility Index (VI), VCI, visible length, and Collision Risk Index (CRI) are image-based operational measurements of the same detectability mechanisms under standardized UAV acquisition conditions. Here we address this gap using UAV imagery and image-derived metrics, focusing on the wintering grounds of the Black-necked Crane on the Tibetan Plateau. Specifically, we aim to: (1) quantify how warning sleeves and warning balls alter power-line detectability; (2) test how key contexts (weather, observation distance, viewing angle, and flight/observation height) shape detectability; and (3) provide decision-ready outputs for conservation planning, including context-matched mitigation comparisons and span/segment prioritization.

We treat the Black-necked Crane wintering grounds on the Tibetan Plateau as a model system for collision-risk mitigation because (1) background visibility conditions shift strongly with snow cover, cloud, and illumination, providing a stringent “stress test” for detectability metrics; (2) observation geometry can be controlled through stratified sampling across distance, viewing angle, and flight/observation height; and (3) the management question is clear and generalizable—prioritizing hazardous spans and selecting effective marking strategies. Importantly, our goal is not to propose universal thresholds, but to offer a standardized, context-calibrated workflow that can be adapted to other species, habitats, and linear infrastructures only after re-estimating context-specific detectability gains under matched conditions.

## Materials and Methods

### Study area

This study focused on three typical wintering grounds of the Black-necked Crane, located in the Tibet Autonomous Region: Linzhou County, Mozhugongka County, and Lazi County in Shigatse. These areas are located in the central Tibetan Plateau, within the Lhasa River basin and the upper reaches of the Nianchu River. They are characterized by high-altitude wetlands and agricultural ecosystems. These sites serve as important roosting and foraging areas for the Black-necked Crane during winter. The study area mainly consists of plateau mountain basins, with an average altitude of approximately 3,900–4,300 m. The winter climate is cold and dry, with strong winds. Vegetation mainly consists of alpine meadows and farmland, and numerous water channels and ponds offer food and roosting resources for the Black-necked Crane. Winter counts and local monitoring records indicate that these sites collectively support a substantial wintering population of Black-necked Cranes, with more than 7,000 individuals recorded during the wintering season.

In recent years, rapid expansion of regional electrical infrastructure has introduced 35 kV, 110 kV, and 220 kV transmission lines into the main wintering landscapes of Black-necked Cranes in Linzhou, Mozhugongka, and Lazi. In this study, 11 representative line segments were surveyed, with a total surveyed length exceeding 50 km; several of these segments intersect core roosting, foraging, and commuting areas used by wintering cranes. Previous satellite-tracking and field investigations have shown that collision with power lines is a major mortality factor for juvenile Black-necked Cranes at Tibetan wintering grounds (Wang et al., 2021). More broadly, collision vulnerability is also well documented for other large-bodied birds, including cranes, swans, and geese, when flight routes overlap with overhead wires (Bevanger, 1998; Bernardino et al., 2018; Jenkins, Smallie & Diamond, 2010). In addition to Black-necked Cranes, the wetland–farmland mosaic in the study region is also used by other large waterbirds such as Bar-headed Geese (*Anser indicus*) and Ruddy Shelducks (*Tadorna ferruginea*), indicating that the detectability framework developed here may have wider relevance for large bird conservation in high-altitude power-line landscapes. These characteristics make the region an appropriate model system for evaluating transmission-line detectability and mitigation performance.

### Facility characteristics and environmental condition setup

To comprehensively evaluate the impact of facility design and environmental conditions on the visibility of power transmission lines in Black-necked Crane wintering grounds, the study established the following experimental variables in power transmission line areas across Linzhou County, Mozhugongka County, and Lazi County.

#### Facility design factors

##### Warning devices.

The installation of warning balls and warning sleeves (categorized as ‘Yes’ and ‘No’) to simulate their effect on different transmission lines.

##### Observation height categories.

Height categories were defined relative to the vertical structure of each power-line span rather than as universal absolute altitudes, because wire heights varied among voltage levels and tower configurations. In the baseline no-device dataset, four height categories were used to represent the main vertical positions from which an approaching bird or UAV camera might view the wires: Height 1, near the lower conductor; Height 2, near the middle conductor; Height 3, near the upper/shield wire where marking devices are usually installed; and Height 4, above the shield wire, representing an elevated approach or descent view against the ground background.

For the device datasets, the design was simplified to two relative height categories because warning sleeves and warning balls were installed on or near the shield wire. Height category 1 represented views from below the marked wire, whereas Height category 2 represented views from above the marked wire. These two categories were therefore not a statistical collapse of the four baseline levels, but a device-specific sampling design intended to compare marker detectability from below- *versus* above-wire viewing contexts. In the Results, we now report all four baseline height categories and explicitly indicate when the two-category device design is being used.

#### Observation conditions

##### Distance factor.

Observation distance. Four distance classes were used: 20, 50, 80, and 100 m. These distances were selected primarily on the basis of repeated field observations of Black-necked Cranes in wintering areas, combined with the practical need to establish a standardized near-to-far sampling gradient for UAV image acquisition. They represent short- to moderate-range encounter distances at which cranes may visually approach, cross, or move near transmission lines during flight, commuting, and foraging-related movements. The purpose of this design was not to define exact behavioural reaction thresholds, but to compare how line detectability changes across a realistic range of observation distances under matched environmental conditions.

##### Weather conditions.

Three typical winter weather conditions were selected. Sunny, cloudy, and snowy, representing the typical changes in lighting and background brightness that Black-necked Cranes may encounter during winter.

##### Viewing angles.

Images were collected at five horizontal angles. 30°, 60°, 90°, 120°, and 150°, covering multiple visual perspectives.

### Image acquisition and processing

To obtain high-precision and standardized image data of the power transmission lines for supporting visibility analysis and the subsequent quantification of visual complexity indices, a DJI Mavic 3 drone was used for systematic aerial photography in typical line areas during the wintering period. The UAV was not maintained at a single fixed altitude above ground across all surveyed spans. Because conductor and shield-wire heights varied among voltage levels and tower configurations, the UAV altitude was adjusted at each span to reach the predefined vertical position relative to the wire structure. For the baseline dataset, the camera was positioned near the lower conductor, middle conductor, upper or shield wire, or above the shield wire, corresponding to height categories 1–4. For the warning-device datasets, the camera was positioned below or above the marked wire, corresponding to height categories 1 and 2. Once the target relative position was reached, the UAV maintained a stable altitude during image acquisition. These categories describe standardized image-acquisition geometry relative to the wires and should not be interpreted as exact reconstructions of crane flight altitude. During the flight, photographs were taken to analyze the VI and visible length of the power transmission lines, followed by post-processing to determine the visual complexity. All UAV surveys were conducted for ecological research only, in compliance with applicable local regulations and with field coordination from relevant local management/cooperating units; no personally identifiable human information was collected, and flight procedures were designed to minimize disturbance to birds.

### Image sample size and balanced factorial structure

Within each dataset, UAV image acquisition followed a complete and balanced factorial structure, with one image acquired and included for each unique combination of the predefined factor levels.

The baseline no-device dataset comprised three weather categories (sunny, cloudy, and snowy), five viewing-angle categories (30° , 60° , 90° , 120° , and 150° ), four observation-distance categories (20, 50, 80, and 100 m), and four relative height categories (height categories 1–4). One image was acquired and included for each unique combination of these factors, yielding 3 × 5 × 4 × 4 = 240 images.

The warning-sleeve dataset comprised two weather categories (sunny and cloudy), five viewing-angle categories, four observation-distance categories, two relative height categories, and two sleeve conditions (sleeve absent and sleeve present). One image was acquired and included for each unique combination of these factors, yielding 2 × 5 × 4 × 2 × 2 = 160 images.

The warning-ball dataset followed the same factorial structure as the warning-sleeve dataset, with two weather categories, five viewing-angle categories, four observation-distance categories, two relative height categories, and two ball conditions (ball absent and ball present). One image was acquired and included for each unique combination of these factors, yielding 2 × 5 × 4 × 2 × 2 = 160 images.

The three datasets therefore contained a total of 560 images. Every predefined factor combination within each dataset was represented once. Accordingly, the datasets were fully crossed and balanced, but each factorial cell contained only one observation and therefore had no within-cell replication.

Snowy conditions were included only in the baseline no-device dataset. No warning-sleeve or warning-ball images were acquired under snowy conditions. Therefore, the efficacy of either warning device under snow-covered backgrounds was not evaluated, and the device-related conclusions are restricted to the sunny and cloudy conditions included in the warning-device datasets. The full sample distribution across datasets and factor levels is provided in Table S1.

### Visibility indicator extraction and analysis

**Figure 1:**
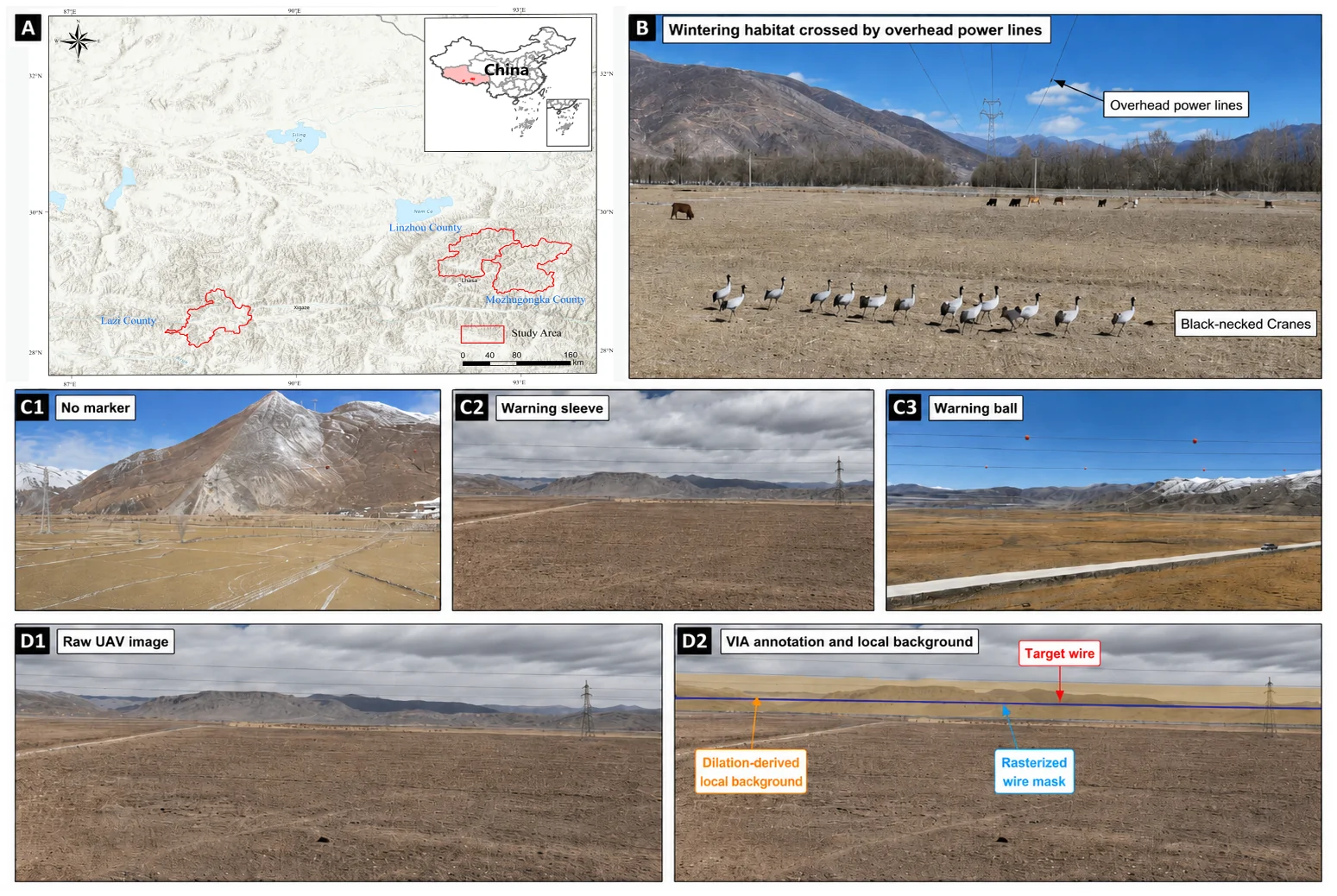
Study area, representative power-line contexts, warning devices, and image-annotation examples. (A) Location of the three Black-necked Crane wintering areas included in this study: Linzhou County, Mozhugongka County, and Lazi County on the Tibetan Plateau. Exact transmission-line locations are not displayed for infrastructure-security considerations. (B) Representative wintering habitat and power-line landscape. (C) Transmission line equipped with warning sleeves. (D) Transmission line without a warning device. (E) Transmission line equipped with warning balls. (F) Representative original UAV RGB image used for image-based detectability analysis. (G) Example of manual polyline annotation using the VGG Image Annotator and the corresponding rasterized transmission-line mask used for subsequent metric extraction.

For the selected UAV images, transmission-line regions were manually delineated using the VGG Image Annotator (VIA; Dutta & Zisserman, 2019), an interactive annotation tool, rather than an automated deep-learning segmentation model. Polyline annotations of the target wires were exported as JSON files, and the annotation coordinates were rasterized in OpenCV to generate binary transmission-line masks for subsequent image analysis. No convolutional neural network, U-Net backbone, model training, or regression loss function was used in this step. Therefore, network-based segmentation metrics such as training IoU are not applicable to this workflow. The masks were visually checked after rasterization to ensure that the annotated line regions corresponded to the target wires before calculating contrast, edge strength, visible length, VI, VCI, and CRI. Representative UAV images from the baseline, warning-sleeve, and warning-ball datasets, together with an example of the VIA-based annotation and mask-generation procedure, are presented in Fig. 1. Seven representative original-resolution UAV images with sensitive location, flight-telemetry, altitude, and equipment-identification metadata removed are provided in Data S1. This process included extracting location and shape information of each power transmission line. All UAV photographs were acquired at a native resolution of 5,280 × 2,970 pixels and were processed at this original resolution. No resizing, stretching, or aspect-ratio modification was applied before image-metric extraction. Images were read in OpenCV, which stores colour channels in BGR order, while the original pixel dimensions were retained throughout the analysis.

To calculate the grayscale-intensity contrast between the transmission line and its local background, each image was first converted to grayscale. A local background neighbourhood was then generated by dilating the rasterized transmission-line mask with a 100 × 100-pixel elliptical structuring element in OpenCV and excluding the original wire-mask pixels. This procedure produced an approximately 50-pixel surrounding neighbourhood around the annotated wire mask. The neighbourhood size was used as a fixed operational window to capture the immediate RGB background while limiting the inclusion of more distant scene elements, and it was held constant across all images to ensure comparability. It should not be interpreted as a species-specific perceptual distance and should be recalibrated when the workflow is applied to images with different resolutions, sensors, or acquisition geometries. The local contrast defined in this study was calculated as follows:

\begin{eqnarray*}Contrast= \frac{ \left\| \overline{{I}_{line}}-\overline{{I}_{bg}} \right\| }{\overline{{I}_{line}}+\overline{{I}_{bg}}+} \end{eqnarray*}

\begin{eqnarray*}=1{0}^{-6} \end{eqnarray*}

where $\overline{{\mathrm{I}}_{\mathrm{line}}}$ and $\overline{{\mathrm{I}}_{\mathrm{bg}}}$ represent the mean grayscale intensities of the transmission-line region and its surrounding local-background region, respectively, and *ɛ* = 10^−6^ is a small constant used to prevent division by zero.

For overall sharpness (clarity) of the image, Laplacian variance was used to measure image sharpness (Pech-Pacheco et al., 2000). Additionally, Sobel gradients were used to quantify edge strength within the transmission-line mask, and Canny edge detection (Canny, 1986) was used where binary edge extraction was required. The image was first converted to grayscale, and the Laplacian operator was used to calculate the edge response of the entire image. The variance of this response was taken as the sharpness indicator. The Sobel operator was applied to calculate the gradient magnitude of the image, and the gradient values within the transmission line mask (where the mask was set to 1) were summed up to calculate the edge strength (E).

Thus, sharpness measured the overall image clarity, while edge strength focused on the cumulative edge sharpness in the power transmission line pixels. After obtaining the contrast and edge strength, both indicators were normalized (assuming the maximum contrast and edge strength) to ensure the VI remained within a controllable range. The VI developed in this study was calculated as follows:

\begin{eqnarray*}VI=\alpha \cdot Contrast+\beta \cdot Edge~Strength \end{eqnarray*}

where *α* and *β* are the weights assigned to contrast and edge strength respectively. This index allows for a more comprehensive quantification of the visibility of power transmission lines in the image by considering both color contrast and edge sharpness. Representative power-line exposure contexts, UAV image examples, and the VIA-based annotation and mask-generation procedure are shown in Fig. 1.

### Visual complexity indicator extraction and analysis

To quantify background complexity in UAV RGB images, the VCI was introduced as a supplementary image-derived descriptor. VCI was not intended to reproduce the full perceptual experience of Black-necked Cranes. Instead, it was designed to describe the amount of texture uncertainty, structural clutter, and colour variation in the image background that could reduce the optical salience of the target wire in a standardized RGB scene. VCI consists of three components: image entropy, edge density, and colour variance, which characterize background complexity from the perspectives of texture uncertainty, structural complexity, and colour distribution.

Image entropy reflects the degree of dispersion in the grayscale distribution of the image. The higher the entropy value, the more texture variation and uncertainty the image contains. The entropy value is calculated as follows (Shannon, 1948):

\begin{eqnarray*}H=-\sum _{i=0}^{255}{p}_{i}\cdot \log \nolimits 2({p}_{i}) \end{eqnarray*}

where *p*_*i*_ is the proportion of pixels with grayscale value *i.*

Edge density is used to reflect the richness of structural contours in the image. First, the Sobel operator is applied to extract edge features from the image (Kanopoulos, Vasanthavada & Baker, 1988), and then the proportion of edge pixels (those with gradient magnitudes above a set threshold) is calculated. The edge density is defined as follows:

\begin{eqnarray*}ED= \frac{{N}_{edge}}{{N}_{total}} \end{eqnarray*}

where *N*_edges_ the total number of edge pixels, and *N*_total_ is the total number of pixels in the image.

Color variance measures the overall dispersion of color in the image. It is calculated by computing the pixel variance for each of the BGR color channels and averaging them. The formula is:

\begin{eqnarray*}CV= \frac{1}{3} \left( \mathrm{Var}(B)+\mathrm{Var}(G)+\mathrm{Var}(R) \right) . \end{eqnarray*}

To distinguish scene-level and local background effects, two spatial scales were extracted from each image: the full-image region, representing overall scene-level background complexity, and the dilation-derived local background neighbourhood around the transmission-line mask, representing the immediate RGB background against which the target wire was detected. Each type of region was independently calculated for the three indicators, followed by Min-Max normalization. The VCI developed in this study was calculated at both the global and local scales as follows:

\begin{eqnarray*}VCI=\alpha \cdot {H}_{norm}+\beta \cdot E{D}_{norm}+\gamma \cdot C{V}_{norm}. \end{eqnarray*}

This study applied scenario-specific Principal Component Analysis (PCA) to extract the weights of the three indicators within each scenario, as the correlation structure between entropy, edge density, and color variance may significantly differ across various scenarios (Jolliffe & Cadima, 2016). The higher the VCI, the more complex the texture, edges, and color variations in the image, indicating that the visual disturbance around the transmission line is more significant, and birds experience greater recognition difficulty during flight.

The difference between Global VCI and Local VCI can reveal the different mechanisms of “overall background complexity” and “perceptual interference around the target” in terms of visibility impact.

### Construction and application of Collision Risk Index (CRI)

CRI construction (relative detectability index): We integrated VI, background complexity (VCI), and visible line length into a composite detectability-oriented index. Components were first standardized to a common direction (higher = easier to detect; thus VCI was sign-inverted), and then combined using data-driven weights derived from principal component analysis (PCA) within the same acquisition context. Because CRI is a relative index (used for ranking contexts/spans and comparing mitigation options), its values are not constrained to [0, 1], and comparisons focus on differences and proportional gains (ΔCRI) rather than absolute magnitudes. Global *vs.* local CRI: Global metrics were computed on the full image, whereas local metrics were computed within the dilated neighborhood around the line; ΔCRI denotes the global–local disparity under the same context.

The CRI developed in this study was calculated as follows:

\begin{eqnarray*}CRI=aVI+b(1-VCI)+VisibleLength. \end{eqnarray*}

The higher the CRI, the better the visibility of the transmission line under the corresponding facility and environmental conditions, and the lower the potential collision risk. We emphasize that CRI is an image-based, relative proxy for detectability that supports span prioritization and mitigation comparison; it is not intended to estimate absolute collision probability without independent collision or movement validation data. The construction of this index aims to integrate visibility and visual disturbance into a unified scale, providing a quantifiable and comparable risk assessment basis for subsequent spatial risk modeling, facility modification prioritization, and conservation strategy optimization.

### Statistical analysis methods for visibility and visual complexity indicators

For the statistical analysis of CRI, one-way analysis of variance (ANOVA) and Kruskal–Wallis rank-sum tests were used to evaluate differences among multi-level contextual factors, including weather, observation height, distance, and viewing angle. For binary comparisons, such as the presence or absence of warning sleeves or warning balls, independent-sample *t*-tests and Mann–Whitney *U* tests were used to provide both parametric and non-parametric support. For significant multi-level factors, *post hoc* pairwise comparisons were conducted using Tukey’s HSD test.

We did not fit a single full-factorial interaction model across all datasets because the baseline no-device dataset and the two warning-device datasets differed in factor structure. Specifically, snowy conditions and four observation-height categories were available only in the baseline dataset, whereas the warning-sleeve and warning-ball datasets included only sunny and cloudy conditions and two relative height categories. Therefore, cross-environmental interactions such as weather × distance or height × viewing angle were not interpreted as part of a complete factorial design. Instead, device effects were evaluated using stratified comparisons within matched weather, height, distance, and viewing-angle categories. This approach was used to assess whether each warning device produced consistent or context-dependent changes in CRI under comparable acquisition conditions.

### Practical implementation and transferability guide

To facilitate adoption in other systems, we summarize a step-by-step workflow and the minimum requirements for applying the approach to different species and landscapes.

#### Minimum data requirements

(1) A mapped set of spans or line segments (span IDs are sufficient); (2) UAV images acquired under a small set of representative contexts; and (3) basic context labels for each image (weather category, observation distance, viewing angle, and approximate flight/observation height).

#### Sampling recipe (recommended)

We recommend a stratified design that varies at least weather × distance, because these two drivers most strongly shape detectability. When feasible, include multiple viewing angles and two or more flight/observation heights to capture geometry effects and to enable matched comparisons among mitigation devices.

#### Computation steps

(1) Delineate the line target from UAV imagery using the same manual annotation and mask-generation procedure; (2) compute VI to quantify target–background contrast and edge strength; (3) compute VCI to characterize background complexity at both global and local scales; and (iv) integrate these components into CRI as a relative proxy for detectability.

#### Comparing mitigation options

To evaluate sleeves *versus* balls (or other warning sleeves), compare CRI under matched contexts (same weather, distance, angle, and height whenever possible) and report the context-specific gain (ΔCRI) as the effect size.

#### Decision-ready outputs

**Figure 2:**
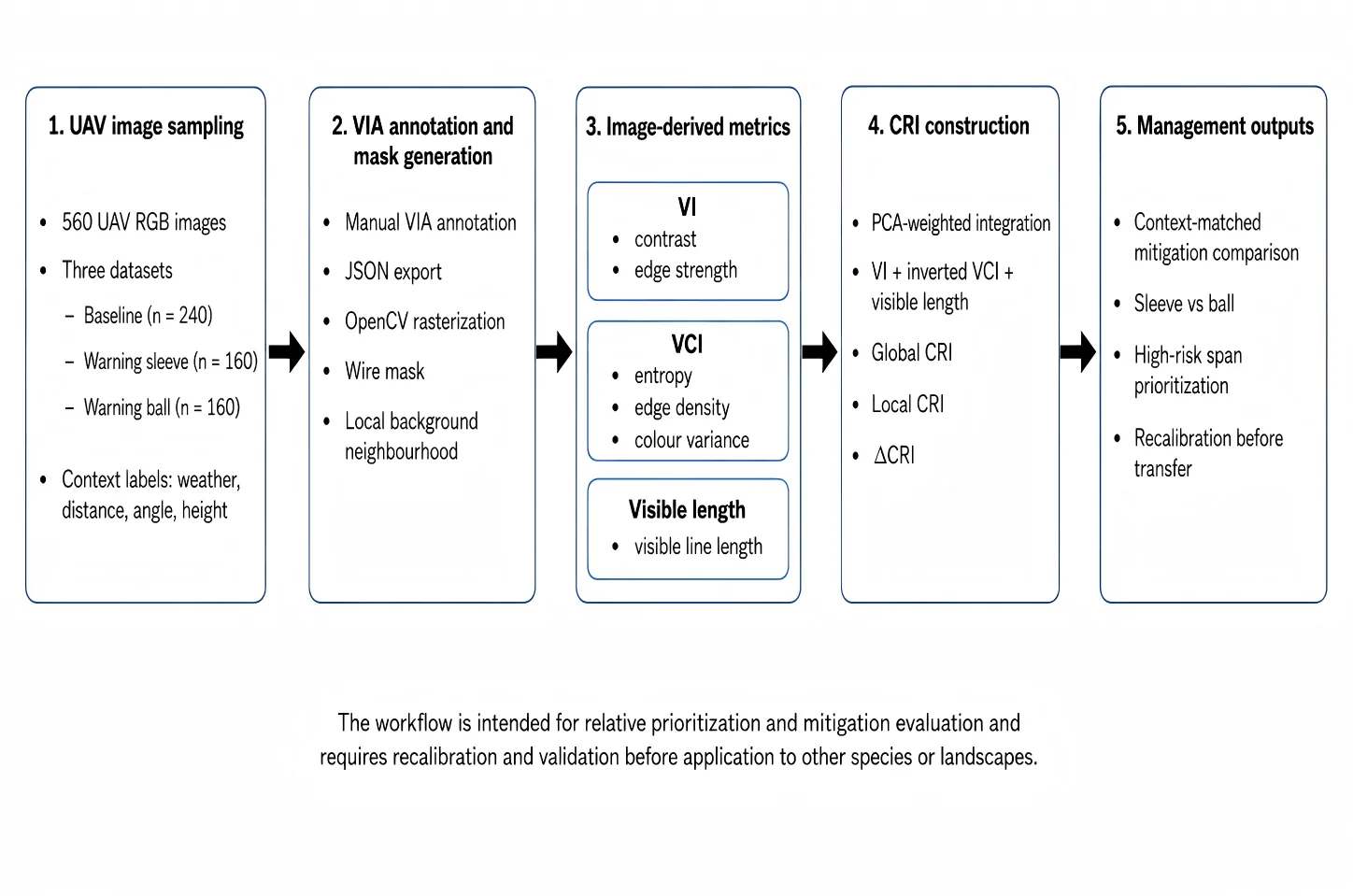
Workflow of the context-calibrated UAV image–based framework for transmission-line detectability and mitigation evaluation. Standardized UAV RGB images were acquired across predefined combinations of weather, observation distance, viewing angle, relative observation height, and warning-device condition. Target transmission lines were manually delineated using the VGG Image Annotator, and the annotations were rasterized to generate binary wire masks. Image-derived metrics were then calculated, including target–background contrast, edge-related visibility metrics, visible line length, image entropy, edge density, and colour variance. These variables were integrated into the Visibility Index, Visual Complexity Index, and Collision Risk Index. The resulting indices were used for context-matched comparisons of warning sleeves and warning balls and for the relative prioritization of power-line spans or segments.

The workflow produces (1) a span-level prioritization list (*e.g.*, average CRI or worst-case CRI across contexts), and (2) a mitigation recommendation indicating which device yields the most robust detectability gains under the dominant high-risk contexts. The complete workflow for UAV RGB image acquisition, VIA annotation, image-metric extraction, CRI construction, and context-matched mitigation comparison is summarized in Fig. 2.

### Ethics, animal welfare, and ARROW compliance

This study adhered to the Animals in Research: Reporting On Wildlife (ARROW) guidelines for wildlife research, the Law of the People’s Republic of China on the Protection of Wildlife, and the Guidelines to the Use of Wild Birds in Research (Fair et al., 2023). Formal institutional animal care and ethics approval was not required because the study was non-invasive and relied exclusively on UAV-based image acquisition of free-living birds and power-line habitats. No birds were captured, handled, tagged, sampled, or experimentally manipulated. Field access and site coordination were conducted with the support of the relevant local management and cooperating units. To follow the 3Rs principles, we used remote observation instead of direct intervention, restricted flight effort to the minimum necessary for standardized image collection, and applied cautious flight procedures to minimize disturbance to birds.

## Results

### Visibility results for the no warning device group (baseline condition)

**Figure 3:**
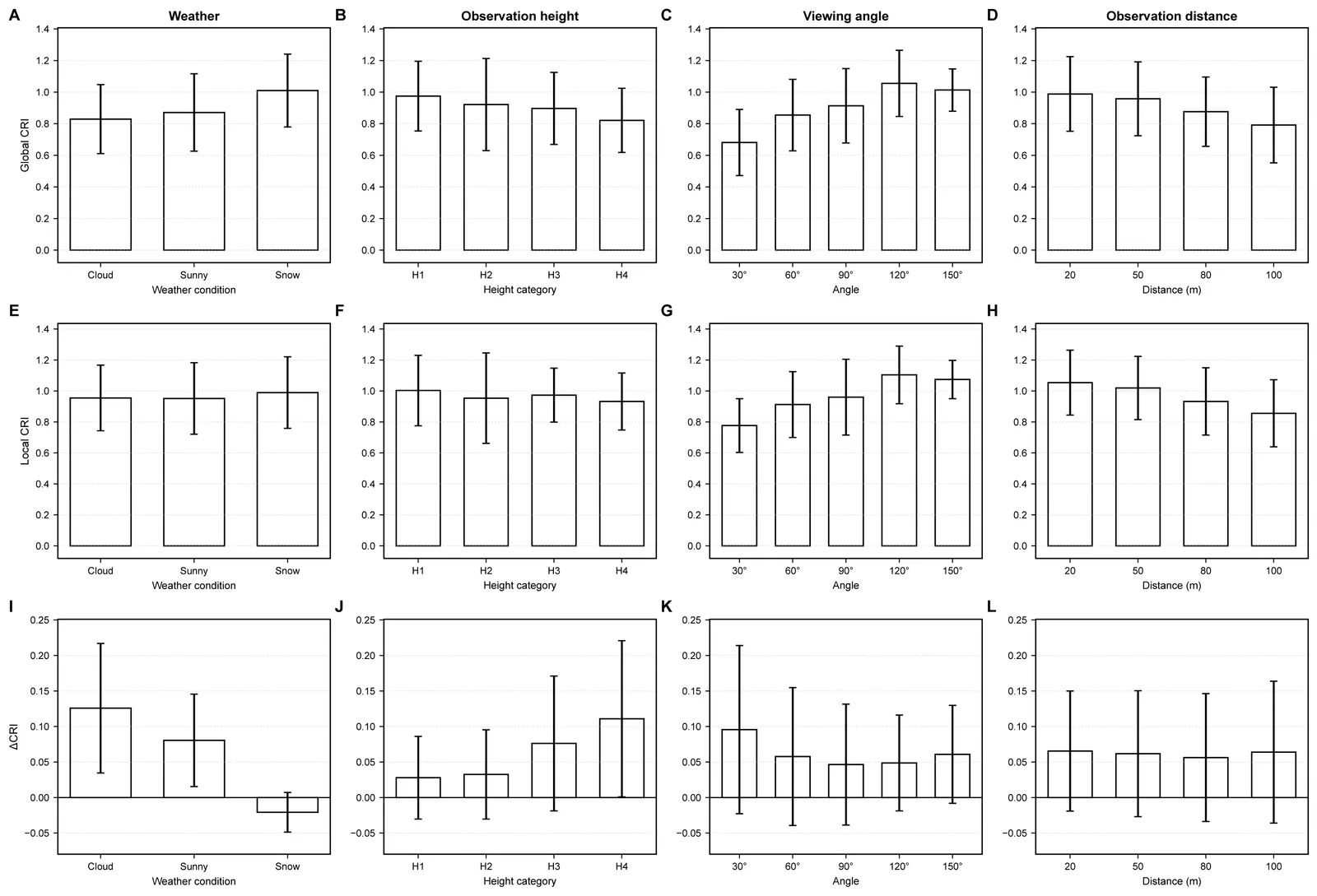
Effects of environmental and observation conditions on transmission-line detectability in the baseline no-device dataset. Global CRI responses to (A) weather, (B) relative observation height, (C) viewing angle, and (D) observation distance; local CRI responses to (E) weather, (F) relative observation height, (G) viewing angle, and (H) observation distance; and global–local CRI differences (Δ CRI) in relation to (I) weather, (J) relative observation height, (K) viewing angle, and (L) observation distance. Values are presented as mean ± SD. Higher CRI values indicate greater image-based transmission-line detectability, whereas Δ CRI represents the difference between global and local CRI under the same acquisition context.

The differences in global visibility of the transmission lines under various weather conditions were significant (ANOVA, *p* < 0.001). In the no warning device condition, the global CRI was highest during snowy weather (1.01 ± 0.23), significantly better than in sunny (0.87 ± 0.24) and cloudy conditions (0.83 ± 0.22; Tukey, *p* < 0.01). The difference between sunny and cloudy conditions was not significant (*p* > 0.05). There was no significant difference in local CRI across weather conditions (*p* ≈ 0.53), but the global-local difference (ΔCRI) was negative during snowy weather (−0.021), significantly lower than in sunny (0.081) and cloudy (0.126) conditions (Tukey, *p* < 0.001). This indicates that snow enhances overall visibility and reduces the disparity in visibility between distant and near parts, thus decreasing the difficulty of recognition due to local detail loss. The corresponding global, local, and ΔCRI weather responses are shown in Figs. 3A, 3E and 3I.

Observation height significantly affected global CRI in the baseline no-device dataset (ANOVA, *p* = 0.0056; Kruskal–Wallis, *p* = 0.0032). Global CRI showed a decreasing trend across the four height categories, from 0.975 ± 0.221 at Height 1 to 0.921 ± 0.292 at Height 2, 0.897 ± 0.228 at Height 3, and 0.821 ± 0.203 at Height 4. The strongest pairwise contrast occurred between Height 1 and Height 4 (Tukey, *p* = 0.0028), whereas the intermediate pairwise comparisons were not significant. Local CRI did not differ significantly among height categories (ANOVA, *p* = 0.359; Kruskal–Wallis, *p* = 0.216). In contrast, ΔCRI increased with height, from 0.028 ±  0.058 at Height 1 to 0.032 ± 0.063 at Height 2, 0.076 ±  0.095 at Height 3, and 0.111 ± 0.110 at Height 4 (ANOVA, *p* < 0.001), indicating that elevated viewing positions increased the gap between scene-level detectability and local line-region recognition. The corresponding global, local, and ΔCRI height responses are shown in Figs. 3B, 3F and 3J.

The horizontal viewing angle significantly affected the visibility of the transmission lines (global CRI: ANOVA, *p* < 0.001). The front view at 30° was the most difficult to recognize (CRI ≈ 0.68), while the view at 120° was the clearest (CRI ≈ 1.06), about 55% better than at 30° (Tukey, *p* < 0.01). There was no significant difference between 120° and 150° angles; both were slightly better than 90° . Local CRI and ΔCRI did not show significant changes with viewing angle (*p* > 0.4), indicating that the angle primarily influenced the overall visibility of the line, while having a limited effect on the clarity of local details. However, some angle-height combinations may still amplify the global-local visibility gap. The corresponding global, local, and ΔCRI angle responses are shown in Figs. 3C, 3G, and 3K.

Observation distance had a weaker but still significant effect on line visibility (global CRI: ANOVA, *p* < 0.001). At 20 m, the global CRI was approximately 0.99, which decreased to 0.79 at 100 m, a reduction of about 20%. The difference between 20 m and distances of 80 m and 100 m was significant (Tukey, *p* < 0.05). Local CRI also decreased with distance (ANOVA, *p* < 0.001), from 1.05 at 20 m to 0.86 at 100 m. Overall, the distance effect was not as pronounced as the effects of weather and angle, and visibility did not deteriorate sharply across the 20–100 m range, suggesting that bird recognition is more limited by the interaction of height and angle. The corresponding global, local, and ΔCRI distance responses are shown in Figs. 3D, 3H, and 3L.

### Visibility results for spans equipped with warning sleeves

**Figure 4:**
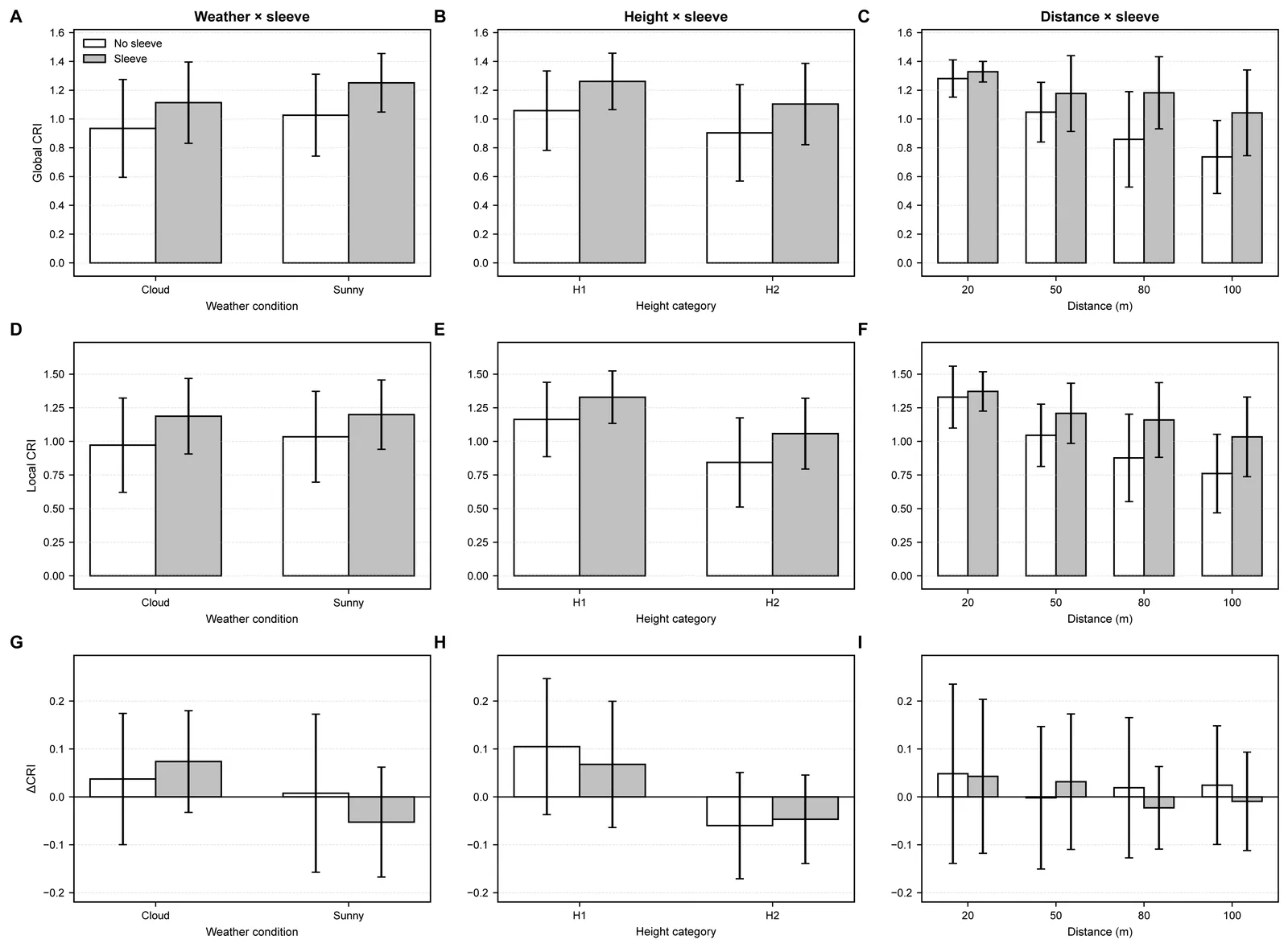
Context-specific effects of warning sleeves on transmission-line detectability. Global CRI comparisons between sleeve-absent and sleeve-present conditions across (A) weather, (B) relative observation height, and (C) observation distance; local CRI comparisons across (D) weather, (E) relative observation height, and (F) observation distance; and corresponding global–local CRI differences (Δ CRI) across (G) weather, (H) relative observation height, and (I) observation distance. Values are presented as mean ± SD. Sleeve effects were evaluated within matched acquisition contexts. Height category 1 represents views from below the marked wire, whereas height category 2 represents views from above the marked wire.

The installation of warning sleeves significantly improved the visibility of the transmission lines and reduced the weather-related differences in visibility. In sunny conditions, the global CRI was approximately 1.03 without the warning sleeves, which increased to 1.25 with the warning sleeves (+22%, Tukey, *p* < 0.01). In cloudy conditions, the global CRI increased from 0.93 to 1.11 (+19%, *p* < 0.05). After installing the warning sleeves, the difference in CRI between sunny and cloudy conditions was no longer significant (1.25 *vs.* 1.11, *p* = 0.13), suggesting that warning sleeves improved visibility under both sampled weather conditions. The corresponding global, local, and ΔCRI weather-specific sleeve effects are shown in Figs. 4A, 4D and 4G.

The installation of warning sleeves improved visibility under both relative height categories. In the below-marker viewing category (height_cat 1), global CRI increased from 1.06 ±  0.28 without sleeves to 1.26 ± 0.20 with sleeves (*t*-test, *p* < 0.001). In the above-marker viewing category (height_cat 2), global CRI increased from 0.90 ± 0.34 to 1.10 ± 0.28 (*t*-test, *p* = 0.005). Local CRI showed the same direction of improvement in both height categories. These stratified comparisons indicate that warning sleeves improved line detectability from both below- and above-marker viewing contexts, although visibility remained lower in the above-marker category because of stronger background interference. The corresponding global, local, and ΔCRI height-specific sleeve effects are shown in Figs. 4B, 4E and 4H.

The visibility gain from warning sleeves became more significant with increasing distance. At 20 m, the improvement was limited and not significant (global CRI 1.28 → 1.33, +4%, *p* = 0.16; local CRI 1.33 → 1.37, +3%, *p* = 0.50). At 50 m, the improvement started to be noticeable: global CRI increased from 1.05 to 1.18 (+12%, *p* = 0.09) and local CRI increased from 1.05 to 1.21 (+15%, *p* < 0.05). At 80 m, the improvement was more significant: global CRI increased from 0.86 to 1.18 (+37%, *p* < 0.01), and local CRI increased from 0.88 to 1.16 (+32%, *p* < 0.01). At 100 m, the effect was the most pronounced: global CRI increased from 0.74 to 1.04 (+41%, *p* < 0.01), and local CRI increased from 0.76 to 1.03 (+36%, *p* < 0.01). There was no significant change in ΔCRI across distances (*p* > 0.1), indicating that the warning sleeves had a similar effect on both global and local indicators. Overall, the further the distance, the more significant the visual gain provided by the warning sleeves, effectively compensating for the insufficient visibility at long distances. The corresponding global, local, and ΔCRI distance-specific sleeve effects are shown in Figs. 4C, 4F and 4I.

### Visibility results for the warning ball group

**Figure 5:**
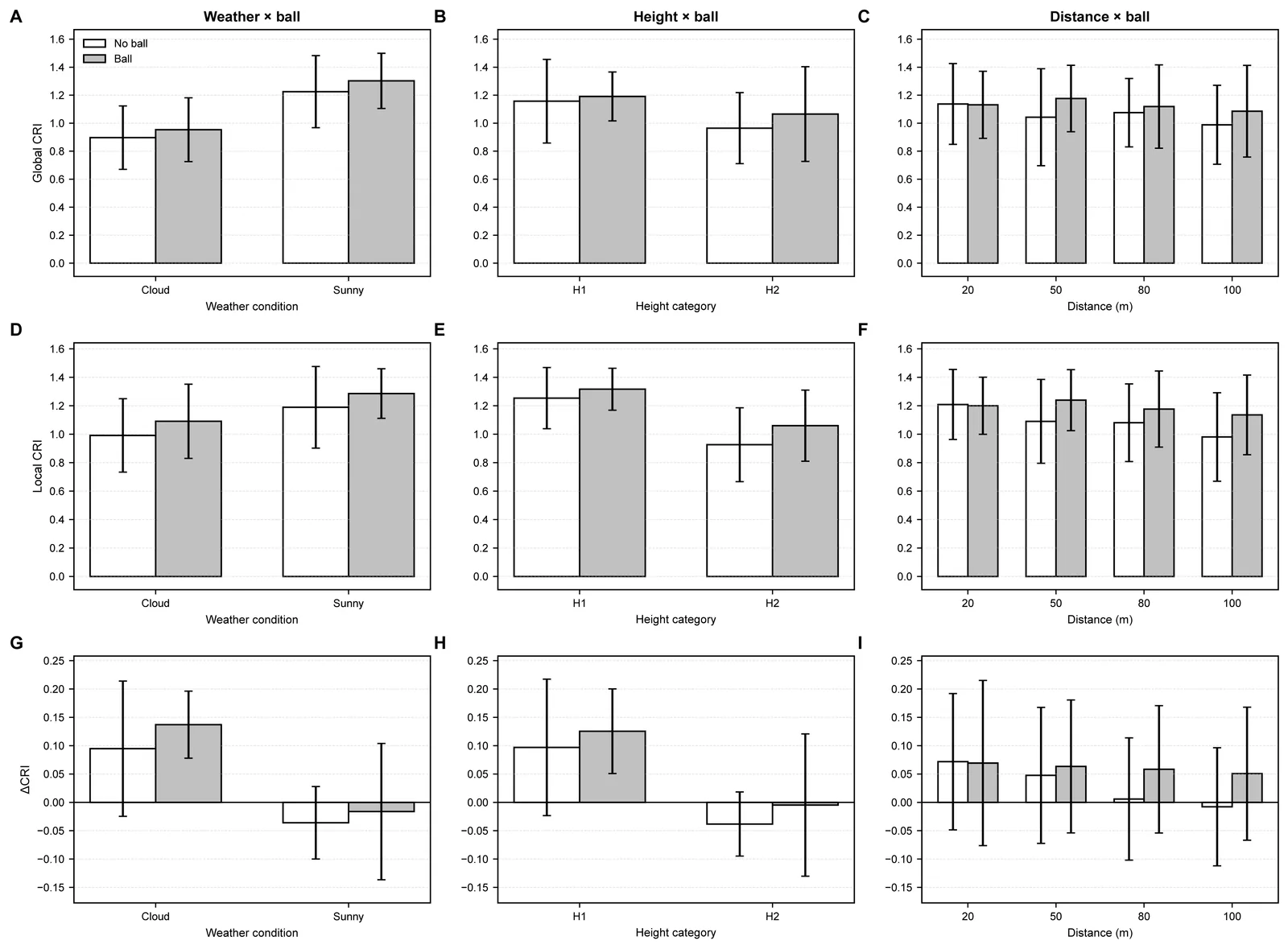
Context-specific effects of warning balls on transmission-line detectability. Global CRI comparisons between ball-absent and ball-present conditions across (A) weather, (B) relative observation height, and (C) observation distance; local CRI comparisons across (D) weather, (E) relative observation height, and (F) observation distance; and corresponding global–local CRI differences (Δ CRI) across (G) weather, (H) relative observation height, and (I) observation distance. Values are presented as mean ± SD. Warning-ball effects were evaluated within matched acquisition contexts. Height category 1 represents views from below the marked wire, whereas height category 2 represents views from above the marked wire.

In cloudy conditions, the warning balls slightly improved visibility: global CRI increased from 0.90 to 0.95 (+6%, *p* = 0.27), and local CRI increased from 0.99 to 1.09 (+10%, *p* = 0.09). However, ΔCRI significantly increased from 0.095 to 0.137 (*p* < 0.05), suggesting that under low-light conditions, local details were more easily recognizable. In sunny conditions, where baseline visibility was higher, the improvement was more limited: global CRI increased from 1.23 to 1.30 (+6%, *p* = 0.14), local CRI increased from 1.19 to 1.29 (+8%, *p* = 0.07), and the difference in ΔCRI was not significant (*p* >  0.3). The corresponding global, local, and ΔCRI weather-specific ball effects are shown in Figs. 5A, 5D and 5G.

In the below-marker viewing category (height_cat 1), the gain was not significant: global CRI increased from 1.16 to 1.19 (+3%, *p* = 0.54), and local CRI increased from 1.25 to 1.32 (+6%, *p* = 0.14). In the above-marker viewing category (height_cat 2), global CRI increased from 0.96 to 1.06 (+10%, *p* = 0.14), whereas local CRI increased from 0.93 to 1.06 (+14%, *p* < 0.05). The difference in ΔCRI was not significant (−0.038 to −0.005, *p* = 0.13). These results indicate that warning-ball effects differed between the two relative observation-height categories, particularly for local CRI. The corresponding global, local, and ΔCRI height-specific ball effects are shown in Figs. 5B, 5E and 5H.

At distances within 20 m, the effect was almost negligible (global CRI ≈ 1.13, local CRI ≈ 1.20, *p* > 0.9). At 50 m, there was a slight improvement: global CRI increased from 1.04 to 1.18 (+13%, *p* = 0.16), and local CRI increased from 1.09 to 1.24 (+14%, *p* = 0.08). At 80 m and 100 m, the increase was 10–16%, but it was not statistically significant (*p* > 0.25). ΔCRI at different distances showed no significant difference (*p* > 0.1). Overall, the warning balls had limited ability to significantly improve line recognition in the 20–100 m range, especially in conditions where the lines were already visible at close range or at specific angles at long distances. The corresponding global, local, and ΔCRI distance-specific ball effects are shown in Figs. 5C, 5F and 5I.

Stratified comparisons by weather, height, and distance showed that warning-ball effects were smaller and less consistent than sleeve effects. Global CRI did not increase significantly within either weather condition, either height category, or any distance class. These results suggest that warning balls provided only limited context-specific improvement in image-based detectability under the sampled sunny and cloudy conditions. The main global CRI responses across baseline, warning-sleeve, and warning-ball datasets are summarized in Table 1.

**Table 1:** Summary of major global CRI responses across baseline and mitigation datasets.

**Dataset**	**Factor**	**Global CRI pattern**	**Statistical summary**	**Main interpretation**
No-device baseline	Weather	Cloud: 0.829 ± 0.218; Sunny: 0.871 ± 0.245; Snow: 1.010 ± 0.231	ANOVA *p*= 3.03 × 10^−6^; Kruskal *p*= 8.22 × 10^−6^	Snow produced the highest global detectability; sunny and cloudy conditions were lower
No-device baseline	Height	H1: 0.975 ± 0.221 H2: 0.921 ± 0.292 H3: 0.897 ± 0.228 H4: 0.821 ± 0.203	ANOVA *p*= 0.0056; Kruskal *p*= 0.0032; H1 *vs* H4 significant	Global detectability declined with observation height
No-device baseline	Distance	20 m: 0.988 ± 0.236 50 m: 0.958 ± 0.234 80 m: 0.876 ± 0.219 100 m: 0.792 ± 0.239	ANOVA *p*= 1.79 × 10^−5^; Kruskal *p*= 9.75 × 10^−5^	Global detectability decreased with increasing distance
Warning sleeve	Weather × device	Cloud: 0.935 ± 0.340 → 1.113 ± 0.282; Sunny: 1.027 ± 0.285 → 1.252 ± 0.204	Cloud: *p*= 0.0126; Sunny: *p*= 1.23 × 10^−4^	Sleeves increased global CRI under both weather conditions
Warning sleeve	Height × device	H1: 1.058 ± 0.276 → 1.261 ± 0.196; H2: 0.904 ± 0.335 → 1.104 ± 0.283	H1: *p*= 3.14 × 10^−4^ ; H2: *p*= 0.0050	Sleeve effects were evident from both below- and above-marker views
Warning sleeve	Distance × device	20 m: 1.281 → 1.328; 50 m: 1.047 → 1.177; 80 m: 0.858 → 1.182; 100 m: 0.736 → 1.043	Significant gains mainly at 80–100 m; 50 m significant by Mann–Whitney test only	Sleeve benefits became more pronounced at longer distances
Warning ball	Weather × device	Cloud: 0.897 ± 0.226 → 0.954 ± 0.228; Sunny: 1.225 ± 0.257 → 1.302 ± 0.197	Global CRI not significant under either weather condition; cloudy ΔCRI increased significantly	Balls produced smaller and less consistent improvements than sleeves
Warning ball	Height × device	H1: 1.157 ± 0.299 → 1.191 ± 0.175; H2: 0.965 ± 0.254 → 1.065 ± 0.338	Global CRI not significant in either height category	Ball effects were weak across height categories
Warning ball	Distance × device	20 m: 1.137 → 1.131; 50 m: 1.043 → 1.176; 80 m: 1.075 → 1.119; 100 m: 0.988 → 1.085	No distance-specific global CRI comparison was significant	Ball effects did not show a stable distance-dependent improvement

## Discussion

This study has verified the three research objectives outlined in the introduction. Overall, our results confirm that warning devices can enhance the visibility of power transmission lines, and significant differences in visibility indices were observed under different observation conditions. Image-based wire detectability was lower under cloudy conditions and at higher relative observation positions. Environmental background and observation conditions significantly modulate the visibility of power lines to birds. The baseline results of this study highlight the mechanisms by which factors such as weather, relative observation height, viewing angle, and distance affect visual recognition.

First, the impact of weather was reflected in changes in background brightness and contrast. We found that during snowy conditions, the overall visibility of the power lines was higher than in sunny and cloudy conditions, which contradicts traditional understanding. It is generally believed that heavy snow and fog reduce the contrast between the power lines and the background, making the lines “blend” into the environment (especially when the background brightness is high and uniform), thus increasing the difficulty for birds to detect them. However, in the context of this study, the snow provided a bright and uniform background, while the transmission lines themselves had a darker color, creating a strong contrast with the snowy background, which made them easier to detect from a greater distance. This also explains why the global CRI was highest in snowy weather, and ΔCRI showed negative values: the overall contour of the line was clearly visible in the long-range view, allowing birds to detect the obstacle without waiting for close proximity. Conversely, in cloudy conditions, the background was gray and cluttered, and the brightness of the transmission lines was similar to that of the background, resulting in the lowest overall visibility (global CRI ≈ 0.83). Birds could only recognize the lines when they were close (ΔCRI was the largest). This result aligns with the views of Bevanger (1994), Bevanger (1998): low-contrast backgrounds greatly weaken birds’ ability to visually detect power lines, which is why collisions occur more frequently in overcast weather. Therefore, increasing the contrast of the transmission lines in poor weather conditions (*e.g.*, using bright colors or luminous materials) is particularly important for reducing collision risks.

Relative observation height affected image-based detectability by changing the background against which the wires were viewed. In the baseline dataset, global CRI decreased from Height 1 to Height 4, whereas ΔCRI increased, indicating a widening difference between scene-level and local detectability at higher camera positions. In the warning-device datasets, height categories 1 and 2 represented views from below and above the marked wire, respectively. These categories describe standardized UAV image-acquisition geometry relative to the wire structure and should not be interpreted as measurements of Black-necked Crane flight altitude or behaviour. The results therefore indicate that vertical viewing context, particularly whether wires are projected against the sky or a heterogeneous ground background, influences image-based detectability. However, they do not directly demonstrate that cranes face greater collision risk at a specific flight altitude or during descent. This interpretation is consistent with evidence that obstacle detection in birds is affected by visual-field orientation and background conditions (Martin & Shaw, 2010; Martin, 2011; Martin, 2012). From a management perspective, warning devices should therefore be evaluated from both below-wire and above-wire viewing contexts, especially where wires overlap visually with complex ground backgrounds.

Observation distance significantly affected image-based line detectability, with both global and local CRI declining from 20 to 100 m. This pattern is consistent with the general expectation that the apparent size and conspicuity of line markers decrease with viewing distance, while marker effectiveness also depends on device design, spacing, environmental background, and the visual characteristics of the focal species (Bernardino et al., 2019; Ferrer et al., 2020; Martin, 2022). In the present study, the stronger sleeve-related gains at 80–100 m indicate that sleeves retained greater optical salience as image scale decreased. However, because the UAV-image analysis did not measure the actual detection threshold or avoidance behaviour of Black-necked Cranes, these results should be interpreted as relative differences in image-based detectability rather than direct evidence of behavioural reaction distance.

This study quantitatively compared the effectiveness of warning sleeves and warning balls in enhancing the visibility of power transmission lines. The results show that both devices can enhance line visibility in various environmental conditions and theoretically reduce collision risks. However, the effectiveness of the two devices varies significantly. Warning sleeves, which continuously cover the lines and have a thicker profile, significantly increase the global CRI by approximately 20% under both sunny and cloudy weather, enhancing brightness and color contrast, making it easier for large birds like Black-necked Cranes to detect obstacles and avoid collisions. In contrast, warning balls showed limited effectiveness in enhancing line visibility at longer distances, especially when the lines were already sufficiently visible at closer distances or at specific angles. This finding is consistent with Chevallier et al. (2015), who reported that warning sleeves, due to their larger coverage area and continuous visual stimulus, are more effective than warning balls. In conclusion, when rapidly reducing bird collision risks is a priority, it is recommended to use warning sleeves as the primary solution, while warning balls can be used as a supplementary measure, especially in low-light or above-marker viewing contexts.

However, the warning-device datasets included only sunny and cloudy conditions. Because no warning-sleeve or warning-ball images were available under snowy conditions, the relative performance of the two devices against snow-covered backgrounds remains unresolved. The higher baseline detectability observed during snowy weather cannot be used to infer marker efficacy under snow, because no matched marked and unmarked comparisons were conducted in that context. Future field sampling should therefore evaluate both devices under snowy conditions using the same matched weather, distance, angle, and height design.

Recent evidence indicates that the effects of energy infrastructure on bird populations vary with infrastructure type and ecological context (Katovich, 2024). Consistent with this broader pattern, our study reveals the combined effects of facility features and environmental conditions on power-line detectability and provides quantitative support for collision-risk management of wintering birds. The results indicate that in high-risk areas such as the wintering grounds of Black-necked Cranes, a single factor alone is insufficient to fully explain collision risks; it is often the combination of multiple unfavorable conditions that leads to the “accident window period.” Therefore, conservation management needs to address these combined effects with targeted measures. First, high-efficiency warning devices should be prioritized in core activity areas of wintering birds. Our data confirms that warning sleeves significantly enhance line visibility, particularly in above-marker and long-distance viewing contexts, while warning balls have relatively limited effects. As such, management departments should consider adopting more prominent, higher-contrast warning sleeves, or adjusting the color of the warning balls to ensure that birds can detect the lines from various distances and angles.

Through an in-depth ecological analysis, this study confirms that improving the visibility of power lines is an effective way to reduce bird collision risks. Warning devices, such as warning sleeves, show promising application prospects in the wintering grounds of Black-necked Cranes. Their effectiveness is modulated by weather, relative observation height, and viewing angle, but remains generally positive. Accordingly, mitigation at high-risk spans should combine site-specific facility optimization with visual marking, accompanied by regular maintenance and evaluation of device performance. More broadly, the long-term sustainability of conservation interventions depends not only on their initial technical effectiveness, but also on local implementation capacity, coordination among responsible stakeholders, continued resource support, and periodic reassessment under changing site conditions (Metananda et al., 2025). A central limitation of this study is that all image-derived indicators were calculated from standard uncrewed aerial vehicle (UAV) RGB imagery. These images represent human-visible optical scenes and do not capture the full spectral sensitivity of Black-necked Cranes, including possible ultraviolet sensitivity or species-specific cone responses. Therefore, VI, VCI, and CRI should not be interpreted as direct reconstructions of crane perception. Nevertheless, visible-spectrum contrast, edge structure, background clutter, and marker conspicuity remain ecologically relevant because avian collision risk is strongly mediated by sensory ecology, visual-field configuration, attention, and the ability to detect static obstacles during flight (Martin & Shaw, 2010; Martin, 2011; Martin, 2012). Our indicators should therefore be interpreted as standardized RGB-based optical proxies for relative detectability, and future studies should incorporate avian visual models, spectral measurements of wires and markers, and behavioural validation to test how well RGB-derived CRI corresponds to crane avoidance responses. A further methodological limitation is that the transmission-line masks were manually delineated using VIA. Although the same annotation protocol and visual mask checks were applied to all images, small annotation errors may affect contrast, edge strength, visible length, VCI, and CRI calculations, especially in low-contrast or visually complex scenes. Future work should quantify this uncertainty through independent re-annotation, inter-annotator agreement assessment, or automated segmentation models validated against manually labelled masks. Additionally, further research combining bird visual models and behavioral response tests could explore the risk levels under different voltage levels and transmission line configurations, increasing the potential for collaborative optimization.

While developed and demonstrated in the Black-necked Crane wintering grounds on the Tibetan Plateau, the workflow is intended to be adaptable to other bird species and landscapes where collision risk is mediated by visual detectability. Transfer should not involve directly exporting the CRI thresholds reported here. Instead, it requires re-estimating the sampling envelope, especially distance and height, to match the focal species’ flight behaviour and likely reaction distances, while retaining stratification by key contexts such as weather and distance. Similar to other composite image-assisted risk indices, broader application requires validation beyond the development dataset before the index can be used for general decision-making (Kamelia et al., 2025). Therefore, future applications of CRI should test its performance across independent landscapes, species, seasons, sensors, and, where possible, observed collision events or behavioural avoidance responses. The approach should be used for relative prioritization and matched mitigation comparison, rather than as a universal threshold or direct estimate of absolute collision probability.

Box 1. Practical guidance for applying the workflow in other systems

(1) Define the unit for action (span/segment) and assign IDs for reporting and prioritization.

(2) Design sampling to capture dominant visibility drivers (minimum: weather × distance; add height/angle when feasible).

(3) Ensure context-matched comparisons when evaluating mitigation devices (same weather, distance, angle, and height as far as possible).

(4) Report decision-ready outputs: (i) ranked priority list (mean and/or worst-case CRI), and (ii) mitigation effect sizes (ΔCRI) by context.

(5) State scope clearly: CRI is a relative, image-based proxy for detectability and is intended for prioritization and mitigation evaluation (not absolute collision probability).

## Conclusion

This study developed a UAV image-based framework to quantify the detectability of overhead power lines in Black-necked Crane wintering grounds on the Tibetan Plateau. By integrating the Visibility Index (VI), the Visual Complexity Index (VCI), and the Collision Risk Index (CRI), we showed that transmission-line detectability is strongly context dependent and varies with weather, relative observation height, viewing angle, and observation distance. Among these factors, poor background conditions and unfavorable viewing geometry can substantially reduce line detectability and increase potential collision risk.

Our comparison of mitigation devices further showed that warning sleeves generally provide stronger and more consistent improvements in detectability than warning balls, particularly under conditions where early detection is most important. In contrast, warning balls produced more limited gains and their effects were more context dependent. These findings suggest that marker performance should not be evaluated in isolation, but under matched environmental and observational conditions.

More broadly, this study shows that UAV imagery can provide a standardized starting point for assessing transmission-line detectability and prioritizing high-risk spans or segments. However, the CRI proposed here should be interpreted as a relative, image-based proxy rather than a direct estimate of collision probability. Future work should therefore focus on three priorities: first, validating CRI against independent collision records, movement trajectories, or observed avoidance responses; second, incorporating species-specific visual models, including ultraviolet sensitivity, to better approximate avian perception; and third, recalibrating and testing the workflow across additional species, seasons, landscapes, and power-line configurations. These steps will be necessary before CRI-based thresholds can be generalized beyond the development datasets used in this study.

## Ethics statement

This study was a non-invasive UAV-based observational study of free-living birds and power-line habitats. No birds were captured, handled, tagged, sampled, or experimentally manipulated, and no personally identifiable human information was collected. UAV surveys were conducted exclusively for ecological research purposes in wetland and power-line habitats used by Black-necked Cranes and other large waterbirds. Fieldwork and site access were conducted with the support and coordination of the relevant local management and cooperating units, and all UAV operations were carried out in accordance with applicable local and national regulations. Flight procedures were designed to minimize disturbance to birds by using controlled flight conditions and cautious field operations. Formal institutional animal care and ethics approval was not required for this non-invasive study.

## Supplemental Information

10.7717/peerj.21732/supp-1 Supplemental Information 1 Distribution of image samples across datasets and experimental factors

10.7717/peerj.21732/supp-2 Supplemental Information 2 Warning ball dataset

10.7717/peerj.21732/supp-3 Supplemental Information 3 Warning sleeve dataset

10.7717/peerj.21732/supp-4 Supplemental Information 4 Weather dataset

10.7717/peerj.21732/supp-5 Supplemental Information 5 Weather analysis code

10.7717/peerj.21732/supp-6 Supplemental Information 6 Representative UAV image of a power line fitted with a warning ball under cloudy conditions

10.7717/peerj.21732/supp-7 Supplemental Information 7 Warning sleeve analysis code

10.7717/peerj.21732/supp-8 Supplemental Information 8 README: Metadata for the example UAV images filenames and associated metadata for the seven example UAV images included in Supplementary Data S1, including experimental category, weather condition, visibility-enhancement treatment, observation distance, viewing angle, height category, camera model, and EXIF-removal status. All geographic coordinates and sensitive geolocation metadata have been removed.

10.7717/peerj.21732/supp-9 Supplemental Information 9 Representative UAV image of a power line fitted with a warning sleeve under cloudy conditions

10.7717/peerj.21732/supp-10 Supplemental Information 10 Warning Ball analysis code

10.7717/peerj.21732/supp-11 Supplemental Information 11 Representative baseline UAV image of a power line without warning markers under cloudy conditions

10.7717/peerj.21732/supp-12 Supplemental Information 12 Representative UAV image of a power line fitted with a warning sleeve under sunny conditions

10.7717/peerj.21732/supp-13 Supplemental Information 13 Representative UAV image of a power line fitted with a warning ball under sunny conditions

10.7717/peerj.21732/supp-14 Supplemental Information 14 Representative baseline UAV image of a power line without warning markers under sunny conditions

10.7717/peerj.21732/supp-15 Supplemental Information 15 Representative baseline UAV image of a power line without warning markers under snowy conditions

## Additional Information and Declarations

### Data Availability

The following information was supplied regarding data availability: The data and code are available in the Supplemental Files.
